# Pressure-induced stromal keratopathy (PISK) from the slit lamp to the optical coherence tomography (OCT)

**DOI:** 10.5935/0004-2749.2024-0322

**Published:** 2025-02-11

**Authors:** Luiz Álvarez Cascos López, Blanca Benito Pascual, Laura Gil Amado, Nabil Dris Hassan, Santiago López García

**Affiliations:** 1 Cornea Department, Ophthalmology Service, Severo Ochoa University Hospital, Leganés, Madrid, Spain

A 46-year-old woman who underwent myopic laser *in situ* keratomileusis (LASIK) and Ahmed valve surgery in the right eye was treated for an infiltrated ulcer in the same eye. After healing and achieving normal central intraocular pressure, she was prescribed topical corticosteroids for corneal haze reduction. Anterior segment optical coherence tomography was performed after 1 month, which showed fluid in the interface between the corneal flap and the stroma, leading to the diagnosis of pressure-induced stromal keratopathy (PISK)^(^1^-^3^)^.

**Figure 1:**
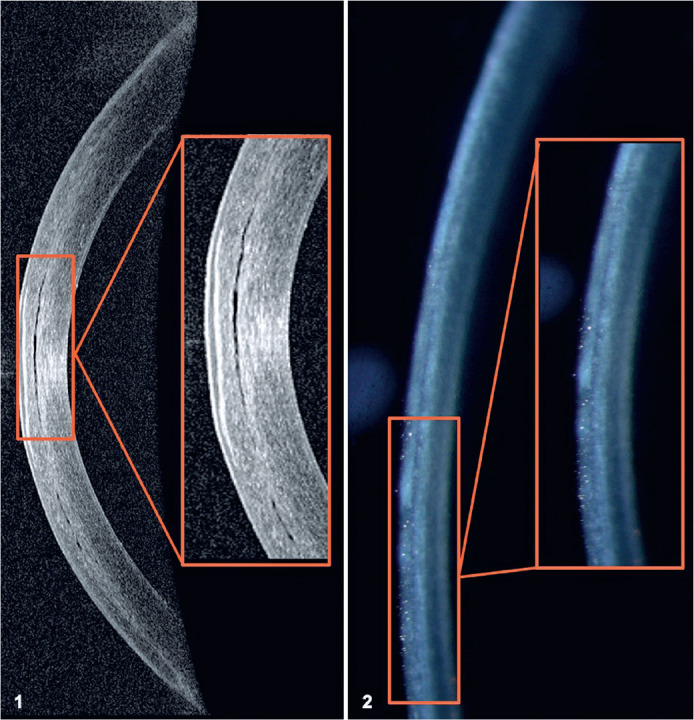
1. OCT image showing PISK; 2. slit-lamp image showing PISK.
